# Mobile phone specific electromagnetic fields induce transient DNA damage and nucleotide excision repair in serum-deprived human glioblastoma cells

**DOI:** 10.1371/journal.pone.0193677

**Published:** 2018-04-12

**Authors:** Halh Al-Serori, Franziska Ferk, Michael Kundi, Andrea Bileck, Christopher Gerner, Miroslav Mišík, Armen Nersesyan, Monika Waldherr, Manuel Murbach, Tamara T. Lah, Christel Herold-Mende, Andrew R. Collins, Siegfried Knasmüller

**Affiliations:** 1 Institute of Cancer Research, Department of Internal Medicine 1, Medical University of Vienna, Vienna, Austria; 2 Center for Public Health, Department of Environmental Health, Medical University of Vienna, Vienna, Austria; 3 Department of Analytical Chemistry, Faculty of Chemistry, University of Vienna, Vienna, Austria; 4 IT ‘IS Foundation, Zurich, Switzerland; 5 Department of Genetic Toxicology and Cancer Biology, National Institute of Biology, Ljubljana, Slovenia; 6 Experimental Neurosurgery, Department of Neurosurgery, University of Heidelberg, Heidelberg, Germany; 7 Department of Nutrition, University of Oslo, Oslo, Norway; Consiglio Nazionale delle Ricerche, ITALY

## Abstract

Some epidemiological studies indicate that the use of mobile phones causes cancer in humans (in particular glioblastomas). It is known that DNA damage plays a key role in malignant transformation; therefore, we investigated the impact of the UMTS signal which is widely used in mobile telecommunications, on DNA stability in ten different human cell lines (six brain derived cell lines, lymphocytes, fibroblasts, liver and buccal tissue derived cells) under conditions relevant for users (SAR 0.25 to 1.00 W/kg). We found no evidence for induction of damage in single cell gel electrophoresis assays when the cells were cultivated with serum. However, clear positive effects were seen in a p53 proficient glioblastoma line (U87) when the cells were grown under serum free conditions, while no effects were found in p53 deficient glioblastoma cells (U251). Further experiments showed that the damage disappears rapidly in U87 and that exposure induced nucleotide excision repair (NER) and does not cause double strand breaks (DSBs). The observation of NER induction is supported by results of a proteome analysis indicating that several proteins involved in NER are up-regulated after exposure to UMTS; additionally, we found limited evidence for the activation of the γ-interferon pathway. The present findings show that the signal causes transient genetic instability in glioma derived cells and activates cellular defense systems.

## Introduction

About 6.8 billion mobile phone subscriptions are active at present (www.itu.int). The adverse health effects of telecommunication radiofrequencies (RF) are controversially discussed since the development of this technology. In 2011, the IARC classified mobile phone RF as “possibly carcinogenic for humans”[1]. This decision was based on results of epidemiological studies which indicated that the RF signals from mobile phones may cause glioblastomas and other malignant brain tumors as well as schwannomas (for reviews see [2, 3, 4]).

It is known that damage of the genetic material plays a key role in the etiology of cancer [5, 6, 7], therefore, we investigated for the first time the effects of the universal mobile telecommunication system (UMTS) signal on DNA stability in human glioblastoma cell lines (U87, U251 and U373). Additionally, we included further human nerve tissue derived cell lines i.e. primary astrocytes, a neuroblastoma line (SH-SY5Y) and a human stem cell like glioblastoma line (NCH421k). We conducted also experiments with cells from organs other than the brain, i.e. liver derived cells (HepG2), buccal mucosa derived and fibroblast cells (TR-146 and ES-1) as well as lymphocytes. All experiments were conducted under conditions relevant for humans (i.e. with specific absorption rate (SAR) values ≤ 1 W/kg) and with a RF-frequency of 1950 MHz. This signal is currently widely used for 3^rd^ generation (“smart”) phones.

The impact of RF on DNA stability was studied in the present investigation in single cell gel electrophoresis (SCGE) assays, which are based on the measurements of DNA migration in an electric field [8, 9]. This approach is currently widely used in genetic toxicology [10]. The experiments were conducted under alkaline conditions, which allow the detection of single and double strand breaks (SSBs and DSBs) and apurinic sites [11]. The cells were treated in all experiments additionally with hydrogen peroxide as some earlier studies indicated that the effects of EMF-fields are due to formation of ROS, therefore we wanted to know if they increase the sensitivity of the different cell types towards oxidative damage. Furthermore, we performed γH2AX experiments which reflect DSBs under identical conditions [12]. This method is based on the measurement of phosphorylation of the histone protein H2AX [13].

It was postulated that RF effects are cell cycle dependent, and it was hypothesized that alterations of DNA repair processes may play a causal role [14], but no results from experiments are available which concern the impact of the UMTS signal on DNA stability in non-dividing cells and on DNA repair functions. Therefore, we studied the effects of RF exposure in two selected glioblastoma lines (U87 and U251, which appeared to be more sensitive towards RF-fields as the other cell types) after cultivation under serum free conditions, which leads to cell cycle arrest and reflects the in vivo situation which is characterized by low mitotic activity. Furthermore we investigated the impact of the UMTS signal on the activities of nucleotide excision repair (NER) and base excision repair (BER), which are major repair pathways in mammalian cells [15]. To provide a mechanistic explanation of our results a proteome analysis was conducted to investigate if proteins which are upregulated as a consequence of DNA damage are affected by the signal. About 4000 individual proteins were quantified before and after treatment of the cells. According to our knowledge, the impact of the UMTS signal on protein expression has not been studied in high-throughput experiments.

## Methods

### Chemicals

Low melting point agarose (LMPA) and normal melting point agarose (NMPA) were purchased from Gibco (Paisley, UK). Propidium iodide, hydrogen peroxide (H_2_O_2_), Triton X-100, Trizma base, trypan blue, fetal calf serum (FCS), Dulbecco's Phosphate Buffered Saline (DPBS), Ham's F12, L-glutamine, Pen-Strep, formamidopyrimidine DNA glycosylase (FPG), 4-(2-hydroxyethyl)-1-piperazineethanesulfonic acid (HEPES), ethylenediaminetetraacetic acid (EDTA), Histopaque 1077, bovine serum albumin (BSA), heparin, Trypsin-EDTA, sodium pyruvate, basic fibroblast growth factor (bFGF), epidermal growth factor (EGF), potassium chloride (KCl), ethylenediaminetetraacetic acid disodium salt dihydrate (Na_2_EDTA) and all media for the cultivation of the cell lines were from Sigma-Aldrich (Steinheim, Germany). Cancer stem cell medium (BIT100) came from Provitro (Berlin, Germany); T4 endonuclease V was purchased from Biozym Scientific (Vienna, Austria). The photosensitizer Ro 19–8022, was from Chiron AS (Trondheim, Norway) and Biofreeze came from Biochrom AG (Berlin, Germany).

### Collection and cultivation of human cell lines

The cell lines were collected from different sources and were stored deep frozen. Prior to the experiments they were checked for contaminations with mycoplasmas, bacteria and fungi.

Human derived glioma lines U87, U251 and U373 were provided by the National Institute of Biology (Ljubljana, Slovenia). The cells were cultivated under standard conditions (37°C, humidified atmosphere, 5% CO_2_) in Dulbecco’s modified Eagle’s medium (DMEM) which was supplemented with 10% FCS. The human neuroblastoma cell line (SH-SY5Y) was grown in complete DMEM: Ham's F12 (1:1 mixture) supplemented with 2.0 mM L-glutamine and 10% FCS. The cells were obtained from S. Böhm (Medical University of Vienna, Austria). The glioblastoma stem-like cell line NCH421k was generated in the laboratory of the Department of Neurosurgery (University of Heidelberg, Germany) and was cultured as non-adherent spheres in DMEM-F12 medium containing 1x BIT100, 2.0 mM L-glutamine, 30.0 U/ml Pen-Strep, 1 U/ml Heparin, 20.0 ng/ml bFGF and 20.0 ng/ml EGF. The human diploid fibroblast line (ES-1 cells) was grown in DMEM with 10% FCS. The cells were obtained from A. Pilger (Department of Occupational Medicine, General Hospital of Vienna, Austria). The human buccal cell line (TR-146) came from J. G. Rheinwald (Dermatology Institute of Boston, USA) and was cultured in Dulbecco’s medium with 10% FCS. The human hepatocellular carcinoma cell line HepG2 was provided by F. Darroudi (University of Leiden, Netherlands). The cells were cultured in Eagle’s minimal essential medium (MEM) supplemented with 1.0 mM sodium pyruvate and 10% FCS.

All cell lines were stored in liquid nitrogen. After re-cultivation, experiments were performed up to the 5^th^ to 7^th^ passage. Media were changed every 2–3 days. When the cultures reached 80% confluency, the cells were washed with DPBS, detached with Trypsin-EDTA, centrifuged and sub-cultured.

### Collection and cultivation of primary cells

Normal human primary astrocytes were purchased from iXCells Biotechnologies (Cat#10HU-035, San Diego, USA). Cells arrived as passage 4 and were cultured in Dulbecco’s modified Eagle’s medium (DMEM) supplemented with 10% FCS and EGF. The cell line was stored and re-cultivated as described above.

Peripheral blood samples were collected from three healthy, non-smoking male volunteers without any history of recent disease or exposure to toxic chemical agents. The samples were collected by venipuncture in heparinized tubes (BD, Heidelberg, Germany).

Blood samples were centrifuged (650 *g*, 10 min, 4°C) immediately after collection. Subsequently, the plasma was removed, the cell suspensions diluted with RPMI 1640 and the lymphocytes isolated by gradient centrifugation (800 *g*, 16°C min, 16°C) with Histopaque 1077 in Accuspin tubes (Sigma Aldrich, Steinheim, Germany). The cells were collected and washed twice in RPMI 1640 (332 *g*, 10 min 16°C).

The Ethical Committee of the Medical University of Vienna specifically approved this study.

### Exposure system

The waveguide-based and computer-controlled exposure system was built and provided by the IT’IS Foundation (Foundation for Research on Information Technologies Society, Zurich, Switzerland, www.itis.ethz.ch). A detailed description of the experimental set-up and of the dosimetry can be found in an article of Schuderer et al. [16]. The exposure unit consists of two rectangular waveguides operating at a frequency of 1950 MHz. Both waveguides were placed in a commercial incubator (HeraCell 240 CO_2_ incubator, Kendro Laboratory, Germany), which provided the environmental conditions for the cell cultures (37°C ± 0.1°C, 5% CO_2_, 95% humidity). A commercial broadband coax-to-waveguide coupler was used to excite the waveguides, each one containing up to six 35 mm ø Nunc Petri dishes (Nunc, Roskilde, Denmark). The detailed electrothermal analysis of the waveguide exposures included both, numerical estimation and experimental validation. The field level was controlled via monopole antennas in the waveguides and linked to the corresponding SAR and temperature values from the dosimetric assessment. The Petri dishes were placed in the H-field maxima of the standing-wave within the waveguide, such that the highest SAR level occurred at the bottom-layer of the dishes, where the monolayer cells are located. The dishes were filled with 4.0 ml medium. A SAR value of 1.0 W/kg, (the maximum intensity which was used in the present investigation) causes under these conditions an increase of the temperature ≤ 0.03°C [16]. The pH values of the cultures were identical in treated and untreated cultures. The system was fully computer controlled and logs the status of the field-level, environmental temperature, and ventilators every 10 seconds for a fully reproducible experiment. Exposure to the different intensities followed the same UMTS protocol at a 5 minutes on/10 minutes off schedule.

### Treatment of the cells

5 x 10^5^ cells were seeded into the Petri dishes and allowed to attach to the bottom overnight before exposure. Aliquots of lymphocyte suspensions were immediately exposed after isolation.

Six dishes were exposed simultaneously in a waveguide chamber; in a second chamber, the cells were sham exposed. The cells were treated intermittently (cycles of 5 min on, 10 min off) as it was postulated that intermittent RF-EMF (radiofrequency electromagnetic fields) may be more effective than continuous exposure [17]. In all experiments, RF-exposed as well as unexposed cells were treated with H_2_O_2_.

For investigations of the SAR dependency, the cells were exposed to intermittent SAR levels of 0.25, 0.5 and 1.0 W/kg for 16 hours.

In a second series of experiments, the cells were grown overnight in FCS medium; subsequently, they were kept in serum free medium for 104 hrs and during the exposure phase (16 hrs); all other experimental conditions were identical as in the first series.

### Single cell gel electrophoresis (SCGE) assays

SCGE experiments were conducted under standard alkaline conditions as described by Tice et al. [8]. Briefly, the cells were collected by trypsinization after exposure, their vitality and numbers were determined with the trypan blue dye exclusion test with a Neubauer chamber (LO-Laboroptik GmbH, Germany).

Subsequently, the pellets were resuspended in low-melting agarose (LMA, 0.5%) and spread on pre-coated agarose slides (1.5% normal melting agarose). H_2_O_2_ was used as a positive control. In all series, also non-RF exposed cultures were treated with H_2_O_2_. The slides were immersed in H_2_O_2_ solution (30 μM) on ice for 10 min. After the treatment, the slides were washed with DPBS (2 x 8 min) and lysed in the dark at 4°C for at least 60 min. After 30 min unwinding under alkaline conditions (pH > 13), electrophoresis was carried out for 30 min (300 mA, 25 V). Neutralization was performed twice for 8 min; then the air-dried slides were stained with propidium iodide (20.0 μg/mL).

The percentage of DNA in tail was measured by use of a computer-aided image analysis system (Comet IV, Perceptive Instruments Ltd., Haverhill, UK). Three independent experiments were conducted per experimental point, and three cultures were set up per experiment. From each culture one slide was prepared and 50 randomly distributed cells were evaluated per slide. Hedgehogs were identified visually and excluded from the evaluation. However, the frequencies were recorded and did not exceed 2%.

### Measurement of DNA repair

#### Preparation of the DNA substrate cells

BER and NER activities were analyzed by use of a modified protocol of the SCGE assay according to A Azqueta et al. [18]. To prepare DNA for BER measurements, HepG2 cells were used as an indicator line and were treated with 0.1 μM of a photosensitizer (Ro 19–8022) for 7 min, and irradiated subsequently on ice with a 400 W halogen lamp (SON-TAGRO, Philips, Vienna, Austria) at a distance of 33 cm to induce formation of 8-oxoguanine. For the measurement of NER, HepG2 cells were irradiated with 2 Jm^−2^ UVC (Stratalinker UV Crosslinker, Model 1800, Agilent Technologies, Vienna, Austria) to generate cyclobutane pyrimidine dimers and 6–4 photoproducts. In parallel, also untreated HepG2 were processed. After two washes with DPBS, the cells were detached by trypsinization and centrifuged at 200 g at 4°C for 3 min. The pellet was resuspended in freezing medium, divided into aliquots and frozen slowly to −80°C.

#### Preparation of the cytosolic extracts from exposed cells

After exposure to RF, the cells were washed twice with cold DPBS, trypsinized and resuspended in medium. Subsequently, they were centrifuged at 800 g (4°C) for 5 min and resuspended in cold DPBS at a concentration of 1.5 × 10⁶/ml and again centrifuged (14.000 g, 4°C) for 5 min. Subsequently, the supernatants were gently removed, then the pellets were snap frozen by dropping into liquid nitrogen and stored at -80°C.

#### Determination of DNA repair

Prior to the repair assay, the cytosolic extracts of RF-exposed cells were thawed and the suspensions were diluted with four volumes of buffer B (45 mM HEPES, 0.25 mM EDTA, 0.3 mg/mL BSA, 2% glycerol, pH 7.8) before use. Extracts were kept on ice until use. Substrate cells were placed on pre-coated agarose slides and lysed in the dark at 4°C overnight. Slides were washed with reaction buffer (0.1 M KCl, 0.5 mM Na_2_EDTA, 40 mM HEPES, 0.2 mg/mL BSA, pH 8.0) for 3 × 5 min. For NER measurements additionally MgCl_2_ solution (final concentration 1 mM) was added. Subsequently, 30 μl of the cytosolic extracts were incubated with substrate DNA for BER and NER measurements for 30 min. Reaction buffer was used as a negative control; for BER assays formamidopyrimidine DNA glycosylase (FPG) served as a positive control while T4 endonuclease V was used in NER experiments.

After incubation, the slides were processed immediately according to the protocol for conventional comet assays [19] to measure DNA breaks introduced by the initial incision events of repair. For each experimental point, three gels were prepared and 50 randomly distributed cells were evaluated per gel. All experiments were performed in triplicate.

### Fluorescence-activated cell sorting (FACS, analysis of the cell cycle and γH2AX analysis)

Cell-cycle analyses were performed with a propidium iodide flow cytometry kit (ABCam, 139418, Cambridge, UK) according to the instructions of the manufacturer. A H2AX phosphorylation assay kit (Millipore, 17–344, Vienna, Austria) was used to analyze DNA damage by flow cytometry. At least 10,000 stained cells were analyzed per experimental point in a FACS BD LSR II (Becton Dickinson, Schwechat, Austria) with FACS Diva Software (Becton Dickinson Biosciences, Schwechat, Austria); the flow cytometry gates remained "fixed" in each experimental series according to the controls for each experimental series. All experiments were performed in triplicate.

### Proteome analysis

#### Subcellular fractionation

The analysis was performed as described previously by Bileck et al. [20]. Briefly, the cells were washed and lysed in isotonic lysis buffer (10 mM HEPES/NaOH, pH 7.4, 0.25 M sucrose, 10 mM NaCl, 3 mM MgCl_2_, 0.5% Triton X-100) supplemented with protease inhibitors (pepstatin, leupeptin and aprotinin, each at 1.0 μg/ml; 1.0 mM PMSF) and exposed to mechanical shear stress as described previously after removal of the supernatants. [21] Cytoplasmic proteins were separated from the nuclei by centrifugation at 2300 g and 4°C for 5 min and precipitated overnight with ice-cold ethanol at -20°C. To obtain nuclear proteins, pellets were swelled up for 10 min in 500 mM NaCl and 1:10 diluted with NP-40 buffer for 15 min. For the separation of nuclear proteins, a centrifugation step at 2300 g and 4°C was performed for 5 min. The extracted proteins were again precipitated overnight with ice-cold ethanol at -20°C. All samples were dissolved in sample buffer (7.5 M urea, 1.5 M thiourea, 4% CHAPS, 0.05% SDS, 100 mM DDT) and protein concentrations were determined with the Bradford assay (Bio-Rad-Laboratories, Germany).

#### MS-sample preparation

All protein samples were enzymatically digested using a modification of the FASP protocol as described previously [21, 22]. Briefly, 3 kD MWCO filters (Pall Austria Filter GmbH) were pre-washed using LC-MS grade water (Millipore GesmbH). 20 μg of each protein sample were pre-concentrated onto the filter by centrifugation at 15,000 g for 15 min. Protein reduction using DTT (5.0 mg/ml dissolved in 8.0 M guanidinium hydrochloride in 50 mM ammonium bicarbonate buffer (ABC buffer), pH 8) and protein alkylation using IAA (10 mg/ml in 8 M guanidinium hydrochloride in 50 mM ABC buffer) were performed. Afterwards, 0.1 μg/μl trypsin was added and incubated at 37°C for 18 hrs. Subsequently, trypsin was added again for another 4 hrs. Finally, digested peptide samples were dried and stored at -20°C until further MS analyses.

#### LC-MS analysis

Peptide samples were reconstituted in 5.0 μl 30% formic acid (FA) containing 10 fmol each of 4 synthetic standard peptides and diluted with 40 μl mobile phase A (98% H_2_O, 2% ACN, 0.1% FA), as described previously [22, 23]. LC-MS/MS analyses were conducted using a Dionex Ultimate 3000 nano LC-system coupled to a QExactive orbitrap mass spectrometer (Thermo Fisher Scientific, Austria). 5.0 μl of the peptide samples were pre-concentrated on a 2 cm x 75 μm C18 Pepmap100 pre-column (Thermo Fisher Scientific, Austria) at a flow rate of 10 μl/min using mobile phase A. Afterwards, peptides were eluted from the pre-column to a 50 cm x 75 μm Pepmap100 analytical column (Thermo Fisher Scientific, Austria) at a flow rate of 300 nl/min. Chromatographic separation was accomplished using a gradient from 8% to 40% mobile phase B (80% ACN, 20% H_2_O, 0.1% FA) over 95 min.

All samples were analyzed in technical duplicates. MS scans were achieved in the range from m/z 400–1400 at a resolution of 70,000 (at m/z = 200), whereas MS/MS scans of the 8 most abundant ions were performed using HCD fragmentation at 30% normalized collision energy and analyzed in the orbitrap at a resolution of 17,500 (at m/z = 200).

#### LC-MS data analysis

MaxQuant 1.3.0.5 running the Andromeda search engine and searching against the SwissProt Database (Version 240314 with 16 717 entries) was used for protein identification as well as label-free quantitative (LFQ) data analysis [24]. An allowed peptide tolerance of 25 ppm, a minimum of two peptide identifications per protein, at least one of them unique as well as a maximum of 2 missed cleavages were included as search criteria.

Furthermore, carbamidomethylation on cysteines was set as fixed modification and methionine oxidation as well as N-terminal protein acetylation as variable modifications. Match between runs was performed using a 5 min match time window and a 15 min alignment time window.

All identified peptides and proteins are meeting an FDR < 0.01. Statistical analysis was performed using Perseus version 1.3.0.4 [25]. Therefore, proteins were first filtered for contaminants, reversed sequences and a minimum of three independent identifications per protein. A two-sided t-test with a significance threshold of p < 0.05 was applied to determine significantly up- and down-regulated proteins between control and treated samples.

### Evaluation of the samples

To ensure blinded evaluation a computer program was used, which generated randomized radiation of the cells. The information concerning the exposure of the cultures was only available to an operator located at the IT’IS Foundation in Zurich.

The slides of comet assays were evaluated by an experienced scorer and were cross-checked by a second scorer. The raw data were collected by a scientist who supervised the study and who received also information concerning the treatment of the cultures (*via* e-mail). To ensure adequate handling, the mails were opened in presence of an external controller (not involved in the project), then both data sets (scoring results and information concerning radiation) were immediately forwarded to a statistician. An identical strategy was used in the proteomics and in the γH2AX experiments.

### Statistical evaluation

Each SAR intensity was tested in three independent experiments. In each experiment, six cultures were set up. Three of them were treated with RF and three were sham exposed. From each culture, one slide was prepared. Sample size determination was based on the requirement that a 50% increase of tail intensity should be detected at a two-sided significance level of 5% with a power of 80%. Three experiments with three cultures per experiment are necessary to fulfill this requirement.

From each culture one slide was made and from each slide 50 cells were evaluated in comet assays by use of a computer-aided image analysis system (Comet IV, Perceptive Instruments Ltd., Haverhill, UK). The percentage of DNA in tail (tail intensity) was determined and stored in a computer file separately for each slide. Hedgehogs were excluded from the evaluation. The data from each file were then automatically extracted and the medians of the tail intensities computed for the 50 cells of each slide.

The percentage values of DNA in tail were arcsine transformed (arcsin $\sqrt{\left(\%\;DNA\;in\;tail/100\right)}$) to remove correlations between means and variances. It is well known that means and variances of tail intensities are correlated [11] which violates a requirement of analysis of variance (ANOVA). The arcsine transformation is efficient in removing this correlation [26]. These data were then evaluated by ANOVA using Statistica 12.0 (StatSoft Inc, Tulsa, OK, USA) including a random factor (experimental repetition) and factors for intensity (different levels of SAR) and exposure (sham/real exposure). Comparisons between real and sham exposed slides were done by linear contrasts. Assumptions of homogeneity of variances were tested by Levene’s tests and assumptions of normality of residuals by Kolmogorov-Smirnov tests with Lilliefors’ corrected p-values.

Data from the cell cycle and γH2AX experiments were analyzed using ANOVA and linear contrasts. For all statistical tests p-values below 0.05 were considered significant.

## Results

### Genotoxic responses of different cell lines

**Fig 1:**
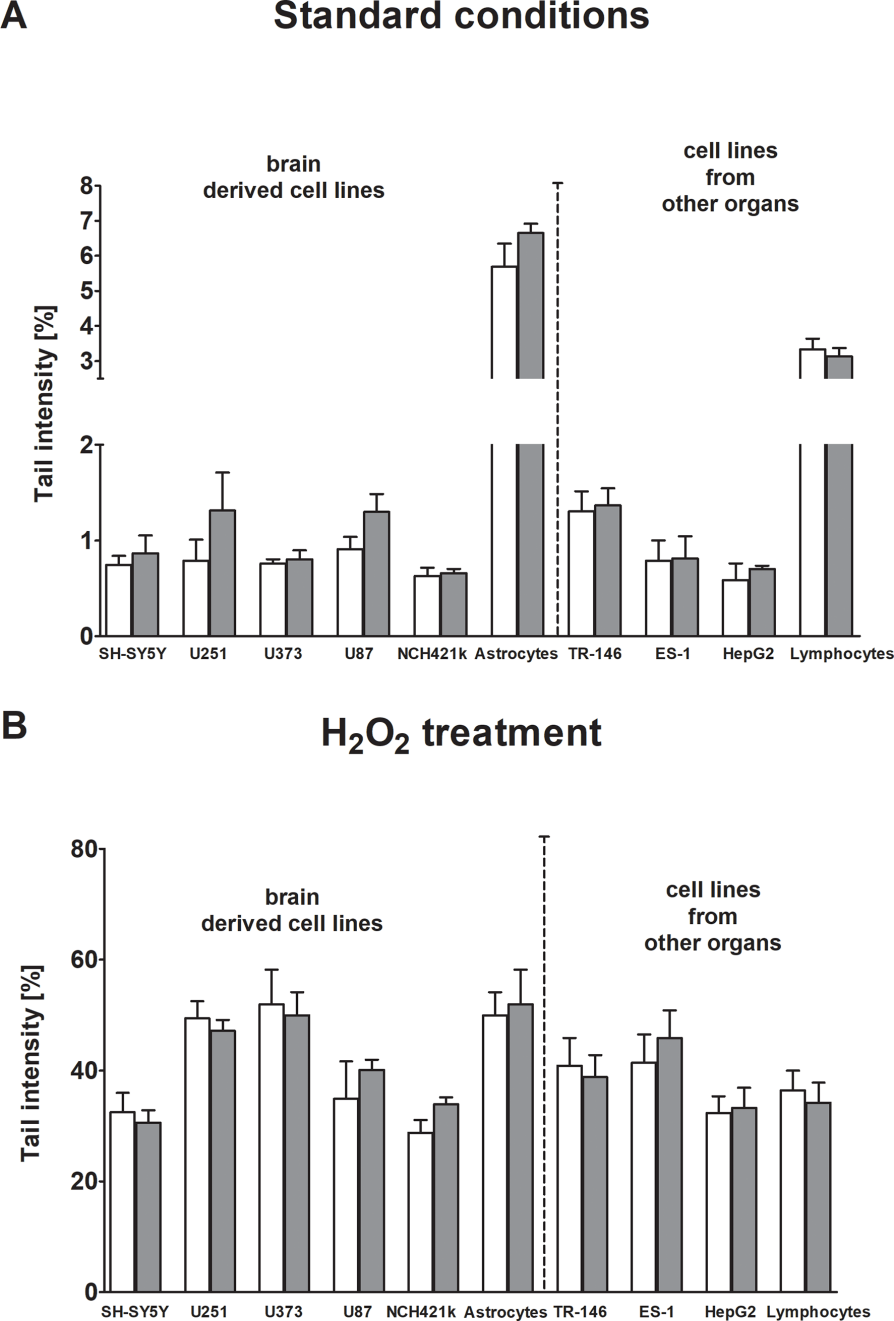
**A and B Impact of RF-exposure on% tail DNA in different human derived cell lines.** The cells were exposed to RF (Specific Absorption Rate 1.0 W/kg) for 16 hrs. Subsequently, some of the cultures were treated with H_2_O_2_ (30 μM) for 10 min as a positive control (Fig 1B). % Tail DNA was monitored in SCGE experiments under standard conditions as described in materials and methods. Bars represent means ± SEM of results obtained in three independent experiments. (Six cultures were prepared per experimental point, three were treated with RF and three were sham exposed. From each culture, one slide was made and 50 cells were evaluated per slide). White bars represent sham exposed cells (controls), grey bars exposed cells. Statistical comparisons were performed by linear contrast after analysis of variance.

The results of screening experiments with the different cell lines exposed to a SAR of 1.0 W/kg in presence or absence of hydrogen peroxide (H_2_O_2_) are summarized in Fig 1A and 1B. Experiments which were performed with lower doses with selected lines are listed in S1 Table. No significant induction of DNA migration was observed after exposure to RF under all experimental conditions. However, an increased level of DNA damage was seen with all neural tissue-derived cell lines after exposure to RF fields; the most pronounced increase in% tail DNA was found with two glioblastoma cell lines U251 and U87 (Fig 1A). Combined exposure to RF fields and H_2_O_2_ was also assessed in all cell lines; the results are summarized in Fig 1B. Exposure to the peroxide caused significant DNA damage in all cell types, but no further increase of DNA damage was detected in RF-exposed cells. The effect of RF-exposure on the vitality of the cells was monitored in all experiments with trypan blue and no cytotoxic effects were detected.

### Impact of serum deprivation on the sensitivity of selected cell lines

**Fig 2:**
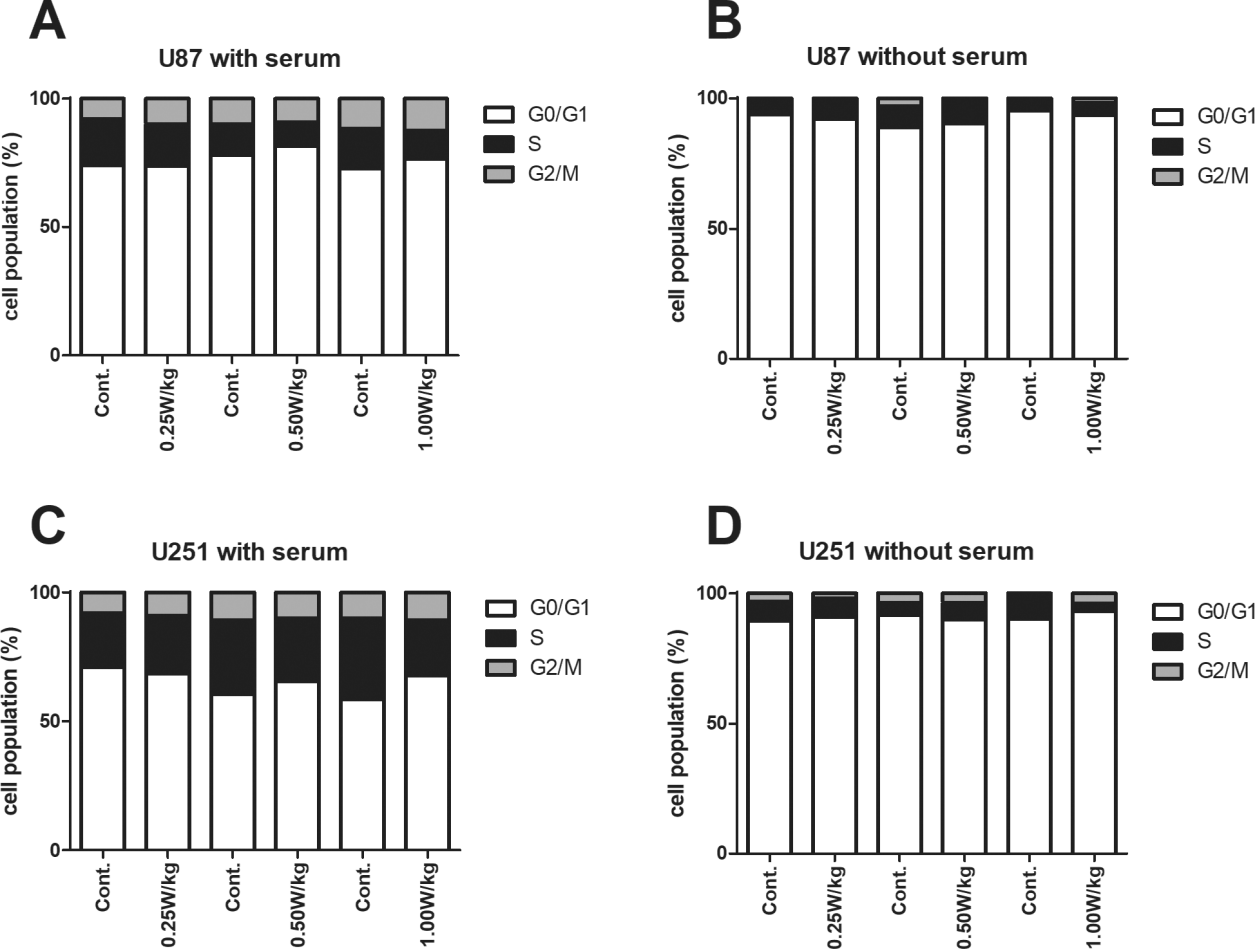
**A-D Impact of the cultivation conditions and RF-exposure on the cell cycle.** The cells were grown in presence and absence of serum and exposed to different RF intensities (Specific Absorption Rates 0.25, 0.5 and 1.0 W/kg) over a period of 16 hrs before they were analyzed by FACS. Cells in G0/G1, S and G2/M phase were sorted on the basis of their DNA contents. 10 000 cells were analyzed per experimental point and all experiments were performed in triplicate.

The most pronounced (although statistically non-significant) increase in% tail DNA was observed after RF-exposure in lines U87 and U251. These cell types were studied in further experiments. It is known that the sensitivity of cells towards ionizing radiation can be increased by serum deprivation [27]. Therefore, we monitored in subsequent experiments the impact of different RF doses on induction of DNA damage in U87 and U251 cells cultivated in absence of serum. Fig 2 shows that serum deprivation had a strong impact on the cell cycle; i.e. when the cells were cultivated with serum, 60–70% were in G0/G1 and 20–40% in S-phase, without serum for 120 hrs, the majority (≥80%) was in G0/G1 and only 8% were in S-phase (Fig 2). RF exposure for 16 hrs had no effect on the cell cycle kinetics of both cell lines (see Fig 2).

**Fig 3:**
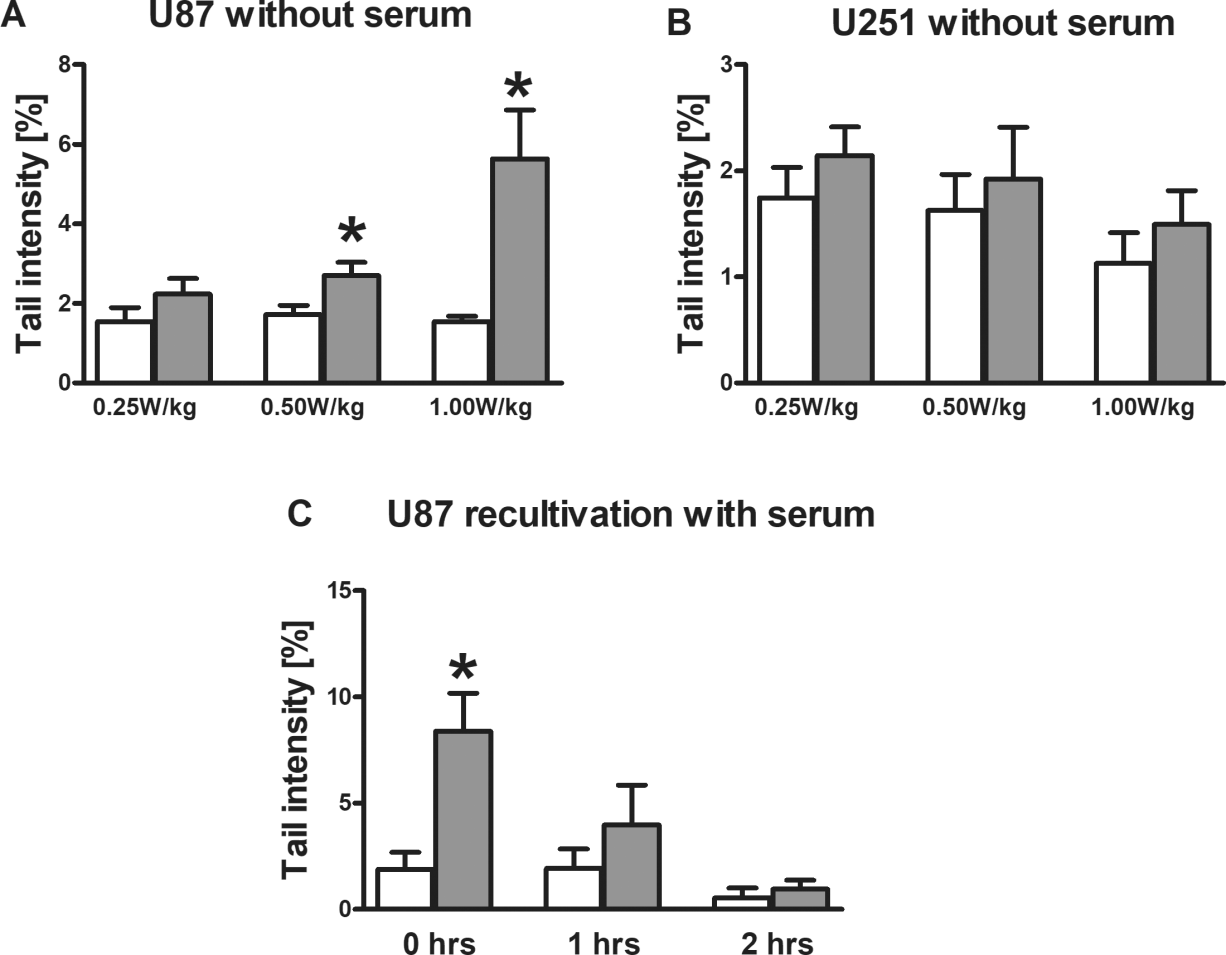
**A-C Impact of RF exposure on DNA stability and vitality of human derived glioblastoma cell lines.** The cells were cultivated in presence or absence of serum and exposed to different RF intensities (0.25, 0.5 and 1.0 W/kg) over a period of 16 hrs. Subsequently, DNA damage was monitored in SCGE experiments under standard conditions (Fig 3A and 3B). Fig 3C shows the disappearance of% tail DNA in U87 cells after RF exposure under serum free conditions and recultivation in serum supplemented medium. The cells were treated with 1.0 W/kg; subsequently, they were cultivated in medium with serum up to two hours. Bars represent means ± SEM of three independent experiments (Six cultures were prepared per experimental point, three were treated with RF and three were sham exposed. From each culture, one slide was made and 50 cells were evaluated per slide). Stars indicate statistical significance (p < 0.05). White bars represent sham exposed cells (controls), grey bars exposed cells. Statistical comparisons were performed by linear contrast after analysis of variance.

When the cells were exposed after serum deprivation to RF-EMF, significant induction of% tail DNA was observed in line U87 while no such effect was detected in U251 (Fig 3A and 3B). The vitality of the cells was in all experimental series above 78% (see S1 Fig); therefore, it can be excluded that cytotoxic effects had an impact on the results of the SCGE experiments.

Fig 3C shows that the “comets” induced in cell line U87 after serum deprivation disappeared after one or two hours, when the cells were re-cultivated in serum supplemented medium.

### γH2AX assays

γH2AX assays were performed to study the induction of DSBs. It can be seen in Table 1 that no significant increases were obtained.

**Table 1:** Impact of RF on γH2AX phosphorylation in human glioblastoma cell lines^1^.

Cell line	SAR (W/kg)	with serum			without serum		
		(mean ± SEM)			(mean ± SEM)		
		Sham exposed^2^	Exposed	p-value	Sham exposed^2^	Exposed	p-value
**U87**	0.25	1.69±0.28	2.14±0.13	0.452	2.49±0.18	2.61±0.15	0.834
	0.5	1.38±0.41	1.39±0.52	0.982	1.83±0.49	1.95±0.41	0.838
	1	2.19±0.13	2.13±0.12	0.914	1.87±0.54	2.12±0.33	0.675
**U251**	0.25	0.83±0.34	1.03±0.11	0.742	1.37±0.63	1.72±0.49	0.556
	0.5	1.31±0.44	1.06±0.20	0.704	2.19±0.41	1.99±0.55	0.742
	1	1.42±0.37	1.54±0.77	0.847	1.31±0.59	1.98±0.39	0.257

^1^ The cells were grown either in serum supplemented medium or in absence of serum. Induction of DNA breaks was monitored in γH2AX experiments (which reflect double strand breaks) after exposure to different specific absorption rates (SAR) for 16 hrs. Values indicate means ± SEM of results obtained in three independent experiments (four cultures were set per experimental point, two were treated with RF and two were sham exposed). Data were analyzed using ANOVA and linear contrasts. For all statistical tests p-values below 0.05 were considered significant.

^2^ Sham exposed cells (controls).

### Induction of BER and NER

**Fig 4:**
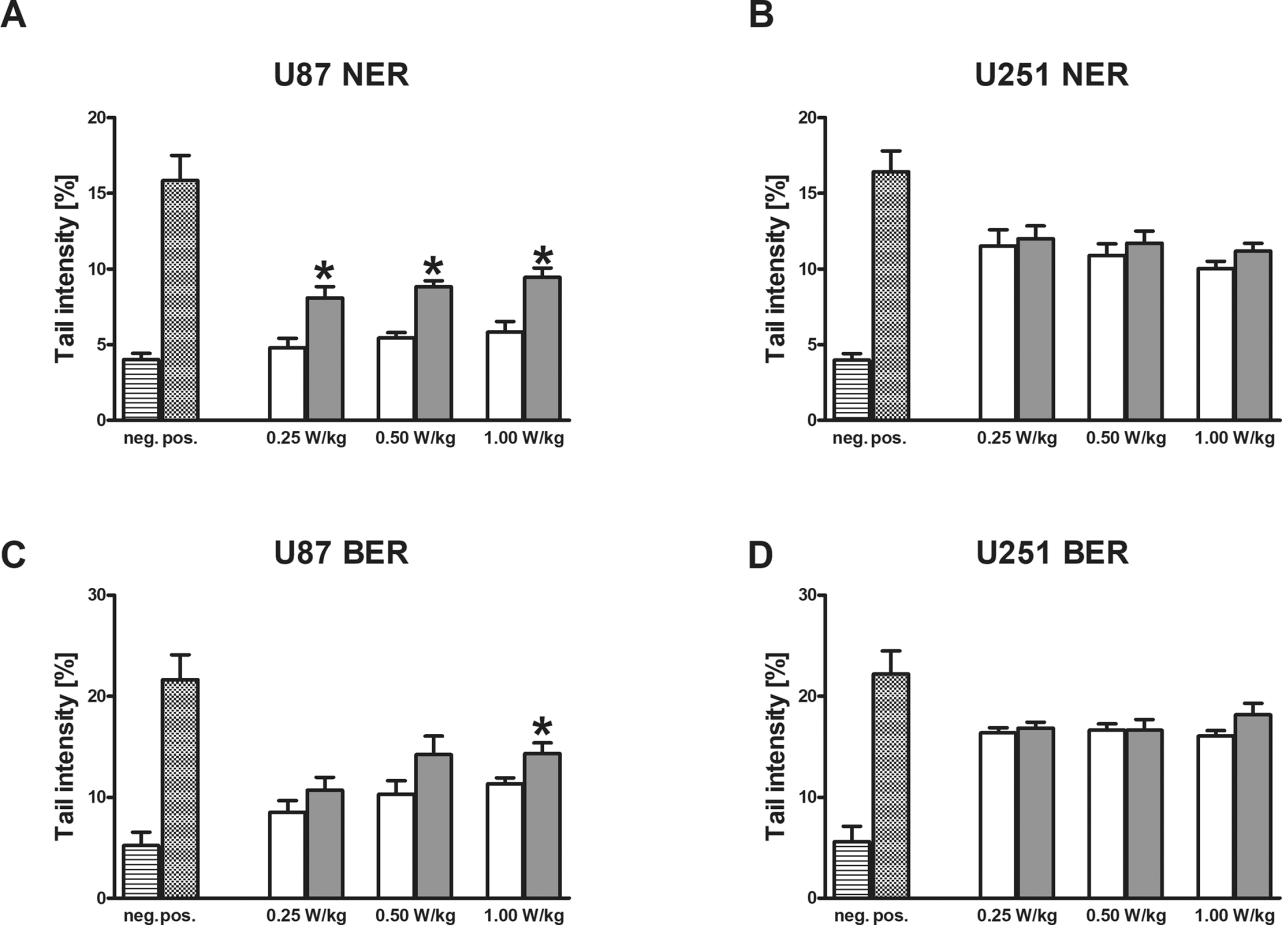
**A-D Measurement of the NER and BER activity in glioblastoma cells after exposure to different RF intensities (Specific Absorption Rate 0.25 W/kg—1 W/kg) for 16 hrs.** Bars indicate means ± SEM of results obtained in the three independent experiments. (Six cultures were prepared per experimental point, three were treated with RF and three were sham exposed. From each culture, one slide was made and 50 cells were evaluated per slide). Left bars show results of control experiments, which were performed in parallel (for details see also Collins and Dusinska [48]). Striped bars show results obtained with isolated nuclei which were exposed to buffer only, hatched bars show results with nuclei which were treated either with UV (for determination of NER) or with Ro-19-8022 (determination of BER). Subsequently, the nuclei of the cells were incubated with T4 endonuclease V (for NER measurements) or FPG (for BER measurements). Other bars show results of experiments in which cells were treated either with UV (determination NER) or with Ro-19-8022 (determination BER) and subsequently with cytosols of sham exposed (white bars) or RF treated (grey bars) glioblastoma cells. Stars indicate significance (p < 0.05). Statistical comparisons were performed by linear contrasts after analysis of variance.

In further experiments which were conducted under identical conditions as the standard SCGE experiments, the impact of RF-exposure on DNA-repair was investigated with a modified version of the comet assay enabling to monitor the activities of NER and BER. The results are shown in Fig 4A–4D. In U87, induction of NER was detected with all SAR intensities (Fig 4A) while U251 was less sensitive (only at the highest dose, a moderate effect was detected). No effects were observed with the latter cell line U251 in BER measurements, but a marginal effect was found in U87 cells with the highest dose.

### Proteome analysis

**Fig 5:**
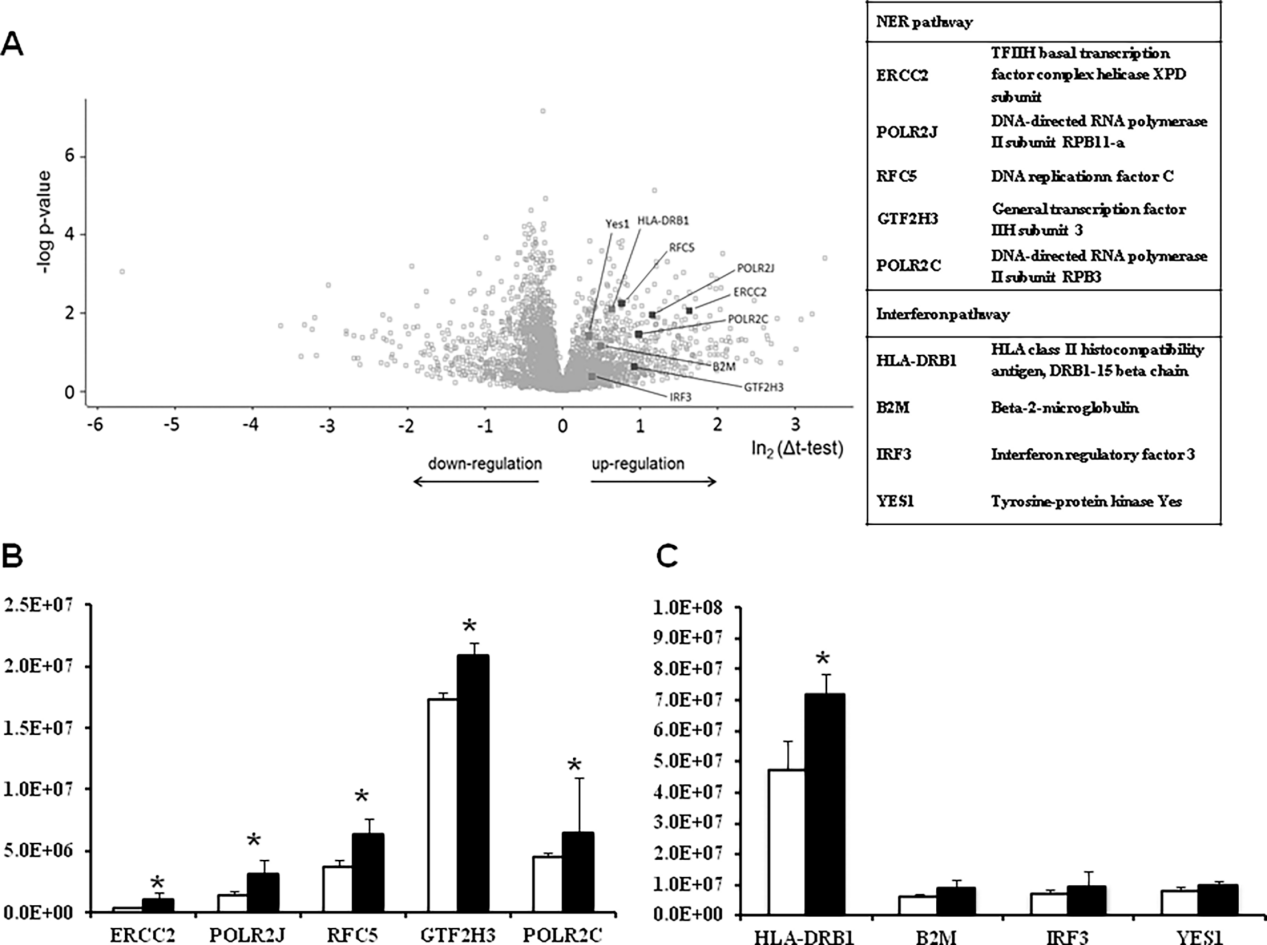
**A-C Results of proteomic analysis of RF exposed glioblastoma cell line U87.** The cells were exposed to RF for 16 hrs (Specific Absorption Rate 1.0 W/kg). The distribution of up- and down-regulated proteins is shown in a volcano plot (Fig 5A). For each identified protein, the fold-change on a logarithmic scale to the basis of 2 (ln_2_Δt-test) and the corresponding p-value (-log p-value) are indicated. Label-free quantification intensities of proteins related to NER activity are shown in Fig 5B and of proteins related to the γ-interferon pathway in Fig 5C. White bars represent sham exposed cells, and grey bars the exposed cells. Stars indicate statistical significance (p < 0.05).

To investigate the impact of RF-exposure on the expression of proteins associated with DNA stability, a mass spectrometry-based proteomic analysis was conducted with U87 cells exposed at a SAR of 1.0 W/kg for 16 hrs. The results are summarized in Fig 5A. In total, 3955 proteins were analyzed. A two-sided t-test between control and RF-exposed cells revealed that the expression levels of 72 proteins were altered. 16 proteins were up-regulated and 56 were down-regulated, respectively (Fig 5A, S2 Table). Fig 5B shows the significant induction of several proteins related to NER. Furthermore, several proteins associated with the γ-interferon pathway were up-regulated (one of them significant) after RF-exposure (Fig 5C).

## Discussion

The present investigation concerns the impact of the UMTS-signal on genomic stability in human derived cells from different organs. Overall, our findings show that this signal presently widely used for mobile telecommunications worldwide does not cause significant induction of DNA damage under “normal” cultivation conditions in any of the ten cell types investigated. However, evidence for formation of comets was found in a glioblastoma cell line under specific experimental conditions (i.e. when cells were cultivated in absence of serum); furthermore, we found clear evidence for induction of a specific form of DNA repair (NER) in this cell line.

As mentioned above, only few studies have been published so far about the effects of the UMTS signal on DNA stability. In three former investigations, SCGE experiments were conducted with lymphocytes from healthy volunteers with consistently negative results (for review see IARC [1] and D Manna, R Ghosh [28]). Furthermore, one article with the human primary fibroblast line ES-1 was published; the experiments were conducted under the same experimental conditions used in the present study [29]. The reliability of these findings was controversially discussed and criticized [30]; furthermore, a German group failed to reproduce these results [31]. Although we found significant but less pronounced increases than Schwarz et al. [29] in a previous experiment applying the same cell line ES-1, in the present experiments three years later the cell line showed no responsiveness to the UMTS signal in accordance with Speit et al. [31].

Induction of DNA damage was observed in the present study only in the U87 glioblastoma line when the cells were cultivated in absence of serum, which leads to cell cycle arrest. In this context, it is notable that earlier experiments with ionizing radiation found that human cells are more sensitive when arrested in G0 phase [32]. This phenomenon was explained by lower levels of protective sulfhydryl compounds in the cytosol at this stage of the cell cycle [27]. The induction of DNA damage which was seen under these conditions may be related to the p53 status of the cells as significant increase of DNA damage was only detected in the p53 proficient line U87. In this context it is notable, that dose dependent induction of NER studied applying a modified protocol of the SCGE assay was only observed in this line. It is well documented that the expression of wild type p53 is involved in the activation of NER [33, 34, 35, 36].

The results of the proteome analysis support the assumption that NER is induced by the UMTS signal as they show that several proteins involved in this repair system are up-regulated as a consequence of RF exposure under serum-free conditions in U87 cells (Fig 5B), namely, ERCC2 a protein belonging the excision-repair cross-complementing family, which is required for transcription initiation by RNA polymerase II [37], two proteins (RPB11-a and RPB-3), which are subunits of RNA polymerase II [38], as well as a DNA replication factor [39, 40] and a subunit of a transcription factor, which are both involved in NER [41, 42]. As shown in Fig 5C we found also limited evidence for activation of the interferon-γ-pathway, i.e. we detected significant up-regulation of the HLA class II histocompatibility antigen (HLA-DRB1), which may mediate cytokine production [43], additionally also some other proteins (B2M, IRF 3, Yes1) were activated which are involved in this pathway, but these latter effects were statistically not significant.

The findings of the γH2AX experiments (Table 1) and also the results of our former MN study with the glioblastoma lines U87 and U251 indicate that the UMTS signal does not cause DSBs and MN which reflect (persisting) chromosomal damage [44].

Taken together, the present findings show that UMTS-modulated RF fields did not cause induction of DNA damage in 10 cell lines in either the presence or absence of H_2_O_2_. Only the U87 cell line under serum-free conditions demonstrated increased DNA damage in response to RF field exposure, where induction of NER appears to be involved. Experiments are in progress to clarify if the activation of NER plays a causal role in regard to synergistic/antagonistic effects which were seen in combined treatment experiments with mobile phone specific radiation and chemicals [45, 46, 47].

## Supporting information

S1 Table Impact of RF-exposure on comet formation in different human glioblastoma cell lines. (DOCX)

S2 Table List of proteins which were altered significantly after exposure to RF in addition to NER-associated proteins. (DOCX)

S1 Fig Impact of radiation exposure on the vitality of the cells which was analyzed with trypan blue exclusion technique. Bars show means ±SD. The experimental setup is described in detail in the legend of Fig 3.
(TIF)

## Abbreviations

BER: base excision repair
DSB: double strand breaks
GSM: global system for mobile communications
H_2_O_2_: hydrogen peroxide
LC: liquid chromatography
MS: mass spectrometry
NER: nucleotide excision repair
γH2AX: phosphorylated histone H2AX
RF: radiofrequency
RF-EMF: radiofrequency electromagnetic fields
SAR: specific absorption rate
SCGE: single cell gel electrophoresis
SSB: single strand break
UMTS: universal mobile telecommunication system
