# Supplementary material for: Mobile phone specific electromagnetic fields induce transient DNA damage and nucleotide excision repair in serum-deprived human glioblastoma cells
Source: PLoS One. 2018 Apr 12;13(4):e0193677. doi: 10.1371/journal.pone.0193677 (PMC5896905; doi:10.1371/journal.pone.0193677)
Supplement: S1 Table — (DOCX) [file pone.0193677.s001.docx]

**Table S1**. Impact of RF-exposure on comet formation in different human glioblastoma cell lines.**^1^**

| **Cell line** | **SAR (W/kg)** | **Standard SCGE (% Tail in DNA)**  **(mean ± SD)** | | | **30µM H_2_O_2_ (% Tail in DNA)**  **(mean ± SD)** | | |
| --- | --- | --- | --- | --- | --- | --- | --- |
|  |  | **Sham exposed^2^** | **Exposed** | **p-value** | **Sham exposed^2^** | **Exposed** | **p-value** |
| **U251** | 0.25  0.50  1.00 | 0.72 ± 0.26  0.57 ± 0.34  0.68 ± 0.37 | 0.73 ± 0.32  0.50 ± 0.06  0.73 ± 0.49 | 0.94  0.64  0.85 | **35.19 ± 12.33**  40.10 ± 4.05  49.41 ± 7.63 | **50.08 ± 9.87**  39.14 ± 10.36  47.15 ± 4.91 | **0.04**  0.84  0.55 |
| **U373** | 0.25  0.50  1.00 | 0.34 ± 0.27  0.52 ± 0.21  0.76 ± 0.10 | 0.47 ± 0.22  0.53 ± 0.43  0.80 ± 0.23 | 0.38  0.96  0.70 | 38.20 ± 7.01  48.98 ± 11.78  51.94 ± 15.32 | 34.15± 5.03  50.29± 1.77  49.98± 10.19 | 0.28  0.79  0.80 |
| **NCH421k** | 0.25  0.50  1.00 | 0.45 ± 0.20  0.48 ± 0.14  0.63 ± 0.21 | 0.69 ± 0.25  0.56 ± 0.18  0.66 ± 0.08 | 0.09  0.59  0.78 | 21.22 ± 6.76  21.74 ± 4.96  28.79 ± 5.09 | 24.26 ± 7.16  21.53 ± 6.40  33.93 ± 2.79 | 0.51  0.95  0.08 |
| **U87** | 0.25  0.50  1.00 | 1.17 ± 0.66  0.65 ± 0.32  1.15 ± 0.32 | 1.81 ± 1.39  1.09 ± 0.38  1.68 ± 0.59 | 0.51  0.20  0.25 | 39.33 ± 9.13  43.46 ± 10.55  47.45 ± 16.33 | 46.77 ± 4.86  37.61 ± 6.33  53.77 ± 6.79 | 0.28  0.46  0.57 |

^1^ The cells were exposed to different specific absorption rates (SAR) for 16 hrs. Subsequently, some of the cultures were treated with H_2_O_2_ (30 µM) for 10 min as a positive control. Comet formation was monitored in SCGE experiments under standard conditions as described in materials and methods Values indicate means ± SD of results obtained in three independent parallel exposure experiments. Six cultures were prepared per experimental point, three were treated with RF and three were sham exposed. From each culture, one slide was made and 50 cells were evaluated per slide. Stars indicate statistical significance (p < 0.05).

^2^ Sham exposed cells (controls).
