# Supplementary material for: Mobile phone specific electromagnetic fields induce transient DNA damage and nucleotide excision repair in serum-deprived human glioblastoma cells
Source: PLoS One. 2018 Apr 12;13(4):e0193677. doi: 10.1371/journal.pone.0193677 (PMC5896905; doi:10.1371/journal.pone.0193677)
Supplement: S2 Table — (DOCX) [file pone.0193677.s002.docx]

**Table S2 List of proteins which were altered significantly after exposure to RF in addition to NER-associated proteins (see Fig. 5B).**

| **Accession** | **Protein name** | **p-value** | **fold-change** |
| --- | --- | --- | --- |
| Q9BYD3 | 39S ribosomal protein L4, mitochondrial | 0.00065616 | 2.3 |
| O96019 | Actin-like protein 6A | 0.00016448 | 1.7 |
| Q02952 | A-kinase anchor protein 12 | 0.00018375 | -1.3 |
| Q6FI81 | Anamorsin | 8.09E-05 | -1.3 |
| P04083 | Annexin A1 | 0.00078335 | -1.2 |
| O95782 | AP-2 complex subunit alpha-1 | 0.00014659 | 1.3 |
| Q2M2I8 | AP2-associated protein kinase 1 | 0.00058898 | -1.2 |
| P18440 | Arylamine N-acetyltransferase 1 | 0.00049593 | 2.5 |
| P25705 | ATP synthase subunit alpha, mitochondrial | 0.00045758 | -1.1 |
| O43491 | Band 4.1-like protein 2 | 5.74E-05 | -1.3 |
| O75531 | Barrier-to-autointegration factor | 0.00091202 | 2.9 |
| Q7L1Q6 | Basic leucine zipper and W2 domain-containing protein 1 | 0.00041666 | 1.5 |
| P11586 | C-1-tetrahydrofolate synthase, cytoplasmic | 0.00096015 | -1.1 |
| P20810 | Calpastatin | 5.19E-05 | -1.2 |
| P30622 | CAP-Gly domain-containing linker protein 1 | 0.00015598 | -1.3 |
| Q8TCG5 | Carnitine O-palmitoyltransferase 1, brain isoform | 0.00039641 | 10.4 |
| Q13098 | COP9 signalosome complex subunit 1 | 0.00041108 | -1.3 |
| P00403 | Cytochrome c oxidase subunit 2 | 0.00065754 | 1.6 |
| P60981 | Destrin | 7.28E-06 | 2.3 |
| Q9NR28 | Diablo homolog, mitochondrial | 0.00056776 | -1.5 |
| Q92466 | DNA damage-binding protein 2 | 0.00064712 | -3.8 |
| P43246 | DNA mismatch repair protein Msh2 | 2.46E-05 | -1.3 |
| P33993 | DNA replication licensing factor MCM7 | 0.00029401 | -1.2 |
| Q9NVH1 | DnaJ homolog subfamily C member 11 | 0.0002088 | 1.7 |
| Q9UJU6 | Drebrin-like protein | 0.0003847 | -1.3 |
| P13639 | Elongation factor 2 | 0.0007002 | -1.1 |
| Q14152 | Eukaryotic translation initiation factor 3 subunit A | 0.00069325 | -1.1 |
| P55010 | Eukaryotic translation initiation factor 5 | 7.26E-05 | -1.2 |
| O75369 | Filamin-B | 1.21E-05 | -1.2 |
| Q14315 | Filamin-C | 0.0004591 | -1.1 |
| P51114 | Fragile X mental retardation syndrome-related protein 1 | 0.00030202 | -1.2 |
| P14314 | Glucosidase 2 subunit beta | 9.54E-05 | -1.3 |
| P04406 | Glyceraldehyde-3-phosphate dehydrogenase | 0.00049707 | 1.3 |
| P08107 | Heat shock 70 kDa protein 1A/1B | 0.00096364 | -1.2 |
| P11142 | Heat shock cognate 71 kDa protein | 0.00033988 | -1.2 |
| P07900 | Heat shock protein HSP 90-alpha | 0.00051372 | -1.2 |
| P51858 | Hepatoma-derived growth factor | 0.00017245 | -1.3 |
| P09429 | High mobility group protein B1 | 6.83E-08 | -1.2 |
| Q16543 | Hsp90 co-chaperone Cdc37 | 0.00034156 | -1.3 |
| Q15181 | Inorganic pyrophosphatase | 0.00012437 | -1.4 |
| Q96AG4 | Leucine-rich repeat-containing protein 59 | 0.0009717 | 1.5 |
| O00754 | Lysosomal alpha-mannosidase | 0.00048886 | 2.3 |
| Q6P1Q9 | Methyltransferase-like protein 2B | 0.00039171 | -1.8 |
| P27361 | Mitogen-activated protein kinase 3 | 0.00080817 | -1.2 |
| P35579 | Myosin-9 | 0.00054219 | -1.3 |
| O14745 | Na(+)/H(+) exchange regulatory cofactor NHE-RF1 | 0.00028502 | -1.5 |
| Q8NF91 | Nesprin-1 | 0.00086446 | -51.1 |
| Q13451 | Peptidyl-prolyl cis-trans isomerase FKBP5 | 6.28E-05 | -1.4 |
| P13797 | Plastin-3 | 0.00020426 | -1.3 |
| Q9UHX1 | Poly(U)-binding-splicing factor PUF60 | 0.00073957 | -1.2 |
| Q8WUM4 | Programmed cell death 6-interacting protein | 0.00043097 | -1.2 |
| P49720 | Proteasome subunit beta type-3 | 0.00014633 | 1.7 |
| Q14320 | Protein FAM50A | 0.00079566 | -1.3 |
| A6NL28 | Putative tropomyosin alpha-3 chain-like protein | 0.00045509 | -1.4 |
| Q13283 | Ras GTPase-activating protein-binding protein 1 | 0.00026596 | 1.4 |
| P46940 | RasGTPase-activating-like protein IQGAP1 | 0.00016359 | -1.2 |
| P52565 | Rho GDP-dissociation inhibitor 1 | 0.00027696 | -1.3 |
| Q12765 | Secernin-1 | 9.69E-05 | -1.4 |
| O00193 | Small acidic protein | 0.00042157 | -1.4 |
| Q14247 | Src substrate cortactin | 0.00029055 | -1.1 |
| P16949 | Stathmin | 0.00073505 | -1.3 |
| Q9UEW8 | STE20/SPS1-related proline-alanine-rich protein kinase | 0.00030621 | 4.2 |
| Q96I99 | Succinyl-CoA ligase [GDP-forming] subunit beta, mitochondrial | 0.00034769 | -1.2 |
| O00186 | Syntaxin-binding protein 3 | 0.00040759 | -1.3 |
| P40227 | T-complex protein 1 subunit zeta | 0.00013445 | -1.3 |
| O14530 | Thioredoxin domain-containing protein 9 | 0.00011888 | -2.0 |
| O43396 | Thioredoxin-like protein 1 | 6.89E-05 | -1.3 |
| Q07157 | Tight junction protein ZO-1 | 0.00045243 | -1.2 |
| P55072 | Transitional endoplasmic reticulum ATPase | 0.00072946 | -1.1 |
| Q5T6F2 | Ubiquitin-associated protein 2 | 0.00029354 | -1.5 |
| Q9C0C9 | Ubiquitin-conjugating enzyme E2 O | 0.00017724 | -1.4 |
| O94888 | UBX domain-containing protein 7 | 0.00097647 | -2.1 |
