# Supplementary figures and images for: Mobile phone specific electromagnetic fields induce transient DNA damage and nucleotide excision repair in serum-deprived human glioblastoma cells
Source: PLoS One. 2018 Apr 12;13(4):e0193677. doi: 10.1371/journal.pone.0193677 (PMC5896905; doi:10.1371/journal.pone.0193677)

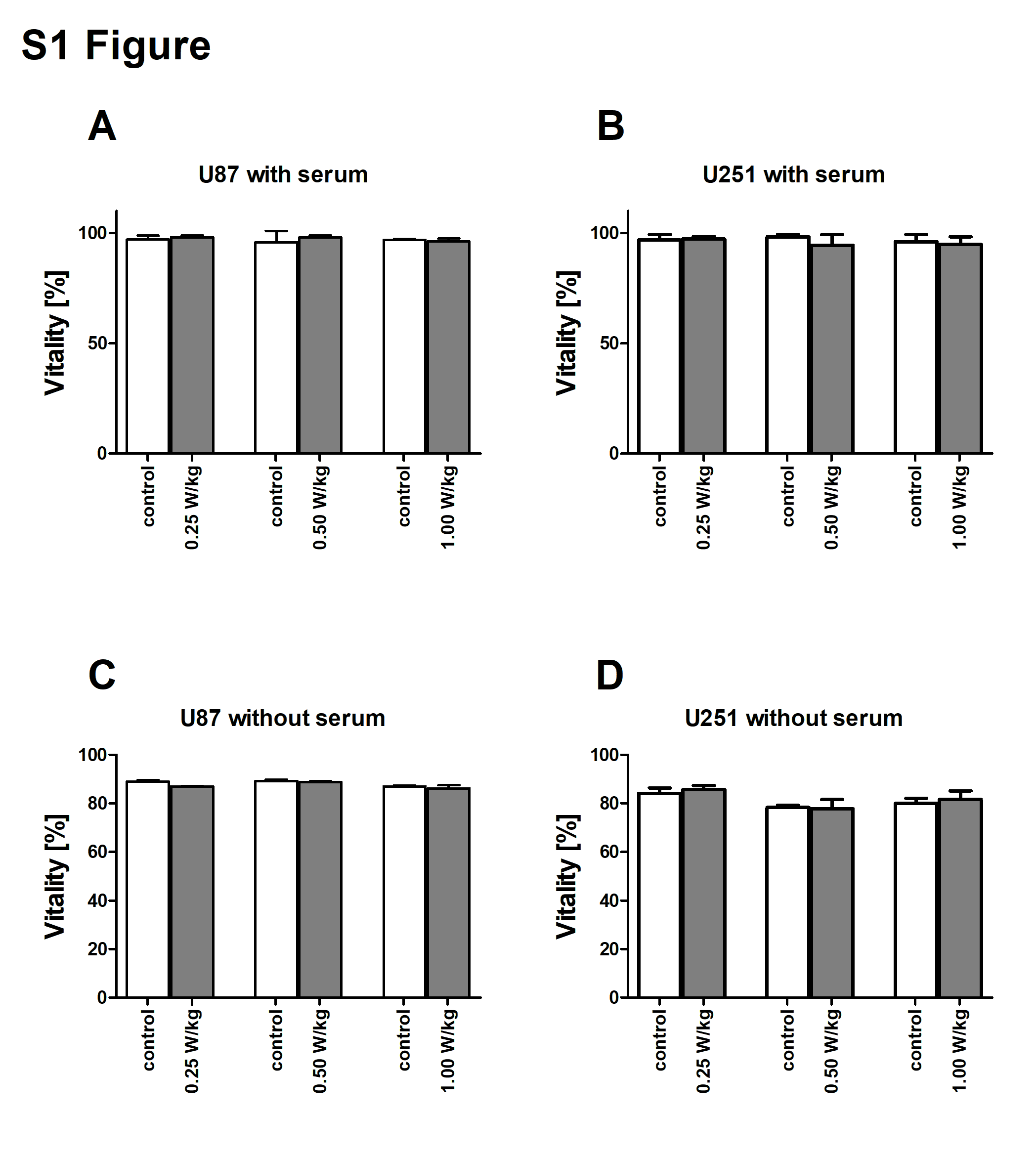

Supplement: S1 Fig — Bars show means ±SD. The experimental setup is described in detail in the legend of Fig 3. (TIF) [file pone.0193677.s003.tif]
